# Fighting against erasure: a community archive of photos of the São Daniel Profeta Church in Manguinhos

**DOI:** 10.1590/S0104-59702023000100072

**Published:** 2023-12-15

**Authors:** Inês El-Jaick Andrade, Éric Alves Gallo

**Affiliations:** i Coordenadora do Núcleo de Estudos de Urbanismo e Arquitetura em Saúde/ Departamento de Patrimônio Histórico / Casa de Oswaldo Cruz / Fiocruz . Rio de Janeiro – RJ – Brasil .ines.andrade@fiocruz.br; ii Arquiteto, Serviço de Educação Patrimonial/ Departamento de Patrimônio Histórico / Casa de Oswaldo Cruz / Fiocruz . Rio de Janeiro – RJ – Brasil . eric.gallo@fiocruz.br

**Keywords:** Favela, Photography, Manguinhos, Favela, Fotografia, Manguinhos

## Abstract

This article describes the construction and development of tangible and intangible ties that link the São Daniel Profeta Church to its community in the territory of Manguinhos by tracing the history of the community’s photo archive through a selection of photographs that were digitized as part of the heritage education project carried out by the São Daniel Profeta Church Preservation Committee between 2019 and 2021. These images improved understanding of the affective relationships which were established and the strategy by the Catholic Church and the government to insert themselves into favela communities during the second half of the twentieth century in the city of Rio de Janeiro.

## Resumo

O artigo apresenta a construção e a valorização dos laços tangíveis e intangíveis que unem a igreja São Daniel Profeta e sua comunidade no território de Manguinhos. Traça a história do acervo de fotografias da comunidade a partir de uma seleção de fotografias digitalizadas no projeto de educação patrimonial desenvolvido pela Comissão de Preservação da Igreja de São Daniel Profeta entre 2019 e 2021. Tais registros fotográficos contribuem para a compreensão das relações de afeto estabelecidas e a estratégia da inserção da Igreja católica e do Estado em territórios de favelas na segunda metade do século XX na cidade do Rio de Janeiro.

A igreja de São Daniel Profeta, um projeto arquitetônico do renomado arquiteto Oscar Niemeyer, está localizada no Complexo de Favelas de Manguinhos, na cidade do Rio de Janeiro, em um contexto socioambiental de implantação bastante diferente de outras construções de sua autoria. Inaugurado em 1960, o templo religioso teve seus atributos formais reconhecidos pelo tombamento estadual em 1966 e a nível municipal em 1998. Ao longo dos anos a "hóstia", como é identificada a igreja pela população local, teve seus elementos estéticos bastante alterados. Apesar disso, permanece como um espaço de identidade e pertencimento para a comunidade, ativando memórias associadas (Gallo, 2021).

A memória ativada coletivamente pode ser descrita como emotiva, vaga, fragmentária, incompleta e tendenciosa em alguma medida (Rossi, 2010). Tal seletividade, que se alimenta de lembranças vagas (Nora, 1993), embeleza o passado em função do presente, de modo consciente ou inconsciente, despertado por um sentimento de nostalgia. Considerando que a memória tem relação direta e afetiva com o passado (Joutard, 2007), no contexto da igreja São Daniel Profeta ela se torna uma ferramenta de coesão comunitária.

Em 2019, a luta comunitária em prol da valorização e conservação desse patrimônio arquitetônico foi reforçada pela criação da Comissão de Preservação da Igreja São Daniel Profeta, um coletivo formado por representantes da comunidade, de universidades e de instituições públicas. A constituição dessa comissão pode ser enquadrada como uma prática insurgente, isto é, seguindo lógica e dinâmica que se configuram, no processo social de confrontação, de baixo para cima (Miraftab, 2016; Gallo, 2021).

Entre as ações desenvolvidas no período de 2019 a 2021 está a primeira Oficina Comunitária: Memórias e narrativas da igreja de São Daniel Profeta, realizada em 24 de novembro de 2019, que culminou na constituição de um acervo fotográfico comunitário, em formato digital, e uma linha do tempo histórica, marcando o início das atividades em comemoração aos sessenta anos de inauguração do templo (1960-2020).

A atividade, sob a perspectiva da educação patrimonial participativa e dialógica (Florêncio, 2014), buscou estimular a construção de lembranças coletivas por meio de rodas de memória entre a comunidade e ancoradas nas fotografias dos acervos pessoais dos participantes. A oficina comunitária contou com aproximadamente 19 pessoas de diferentes idades, entre mediadores, fiéis da igreja de São Daniel Profeta e moradores de Manguinhos. Os resultados foram positivos, no que tange ao envolvimento e à mobilização da comunidade, uma vez que os fiéis se tornaram corresponsáveis por grande parte da comunicação para a realização da oficina.

Na construção do acervo coletivo iconográfico foram digitalizadas cerca de 226 fotografias, distribuídas em oito coleções, cedidas à Comissão com legendas elaboradas pelos cedentes. A construção de uma linha do tempo, de forma linear e fixada ao redor das paredes do templo, permitiu participações para além do dia da oficina assinalada, pois no dia a dia da igreja outras pessoas puderam visualizar e contribuir com a atividade.

A temática das fotos conta a vivência e afetos da comunidade, tendo como mote a igreja e suas atividades litúrgicas, de comunhão e lazer associadas. A atividade revelou histórias da constituição dos acervos, nos quais cada item é representativo de um contexto cultural específico e dotado de significados pessoais. Meneses (1998) reconhece o caráter informativo e documental que os objetos possuem. Os objetos não falam por si, mas trazem consigo uma carga informativa e outra que lhes é atribuída. A fotografia atua como suporte de memória, por meio da sua materialidade, que está relacionada à imaterialidade, como observamos em Manguinhos.

A materialidade é um componente importante para a perpetuação do caráter informativo e documental. Apagamentos, portanto, comprometem e geram rupturas na informação e na transmissão da memória coletiva. Como afirma Rossi (2010), ressurgir de um passado que foi apagado é muito mais difícil que lembrar de coisas esquecidas. "Apagar também tem a ver com esconder, ocultar, despistar, confundir os vestígios, afastar da verdade, destruir a verdade" (p.32). As memórias coletivas fazem parte diretamente do sentimento de pertencimento e, portanto, da construção da identidade comunitária. Nas favelas, historicamente, o apagamento ocorre por diversas causas, como remoções, demolições, enchentes, incêndios, repressões, entre outras. Existe uma conexão direta entre memória e esquecimento, a qual é trabalhada pela historiografia contemporânea.

No contexto de Manguinhos, a partir da coleção sob a guarda de Luiz Antônio de Mello, pode-se relacionar a trajetória de luta contra o apagamento da história da comunidade que tem na igreja o seu referencial de identidade. O conjunto está organizado na forma de álbum fotográfico com aproximadamente oitenta itens produzidos entre 1960 e 1964. Nesse período, esteve à frente da igreja o padre Julius Kremer (1961-1966), a quem se atribui a organização do álbum. É o mais antigo e numeroso conjunto do acervo comunitário recém-constituído. Em primeira análise, seus registros são especialmente significativos porque evidenciam a relação da Igreja em seu contexto territorial, cotidiano, religioso e político. Outro aspecto relevante é a trajetória da coleção ao longo dos anos, desde sua produção até o presente.

Segundo Kopytoff (2008), podemos traçar a trajetória ou biografia dos objetos considerando suas mudanças de função e atribuição de valor. A coleção, organizada em álbum fotográfico, possui páginas estruturadas por assuntos, seguindo uma linha cronológica e com sucintas descrições, conforme as atividades cotidianas e significativas do período: Inauguração da Matriz, 1960; Festa do Padroeiro, 1962; Catecismo, 1962; Romaria a Aparecida do Norte, 1962; Festa do Padroeiro, 1963; Banda dos pequenos jornaleiros, 1963; Rumo a Roma, 1963; Missa Campal, 1963; São Daniel em Marcha, 1963; Passeio Araruama, 1964. Os fatos narrados em imagens sépia revelam o envolvimento da comunidade e a intensidade da comunidade paroquial. São registradas as cerimônias relacionadas diretamente com a comunhão religiosa, como missas, batismos e eucaristia, as festas litúrgicas do padroeiro e a vida social.

O apreço que o padre Julius Kremer tinha por sua paróquia é demonstrado nas legendas em que destaca o aumento da "família paroquial" e descreve a alegria juvenil no passeio do grupo de jovens a Araruama. Também se destaca o deslumbramento na ocasião de sua ida a Roma para participar do Concílio Vaticano II em 1962, durante o pontificado do papa João XXIII (1958-1963). O evento é marcante para a história do catolicismo, pois diversos sacerdotes foram convocados a discutir e regulamentar temas da Igreja católica (1962-1965).

Ao final da década de 1990, o álbum sob a guarda da paróquia de Santa Bernadete (fundada em 1964) foi descartado, junto a diversos documentos, pelo pároco-interventor (1983-1998), José Carlos Guimarães, no processo de transição paroquial. A motivação do descarte não é clara, conforme mencionado por Luiz Antônio de Mello (Gallo, 2021). Antigo morador de Manguinhos e membro da igreja de São Daniel Profeta, na época havia sido proibido de frequentar a paróquia de Santa Bernadete, por sua atuação política marcante, sobretudo no grupo de jovens.[1] Na data do descarte, considerou a documentação importante e, portanto, tendo a possibilidade de resgatar, a salvaguardou. A atribuição de valores distintos ao longo do tempo para o álbum é reflexo das mudanças sociais, políticas e religiosas, desde sua produção até a salvaguarda. Com sua participação na oficina comunitária, compreendendo que o artefato era representativo sobre a memória de sua comunidade, generosamente, cedeu à equipe da comissão para sua reprodução.

Entre as imagens do álbum, a série selecionada para ser apresentada neste artigo é a que se inicia com registros da inauguração da igreja sobre o olhar dos moradores do conjunto habitacional. Diferem-se de fotografias jornalísticas e de imagens produzidas por técnicos do órgão de tutela do patrimônio cultural. A seleção tem o intuito de narrar a estratégia da inserção da Igreja católica e do Estado em territórios de favelas na segunda metade do século XX na cidade do Rio de Janeiro.

## O álbum da igreja de São Daniel: história e resistência

O projeto do templo religioso em forma circular (Figura 1) foi inspirado no modelo canônico de uma hóstia, segundo indicam algumas reportagens da época e relatos de moradores de Manguinhos (Maurício, 25 jun. 1963). A moderna capela de São Daniel, a exemplo de outras obras do arquiteto, como a igreja São Francisco de Assis da Pampulha (1943) e a catedral metropolitana Nossa Senhora Aparecida (1970), foi idealizada por seus patrocinadores para se transformar em uma das maiores atrações turísticas do Rio de Janeiro, apesar de construída em local de difícil acesso. O fato de ela ser circundada por várias favelas já provocava, na época, analogias a história do profeta Daniel, com a máxima que "a igreja São Daniel ficaria rodeada de leões".

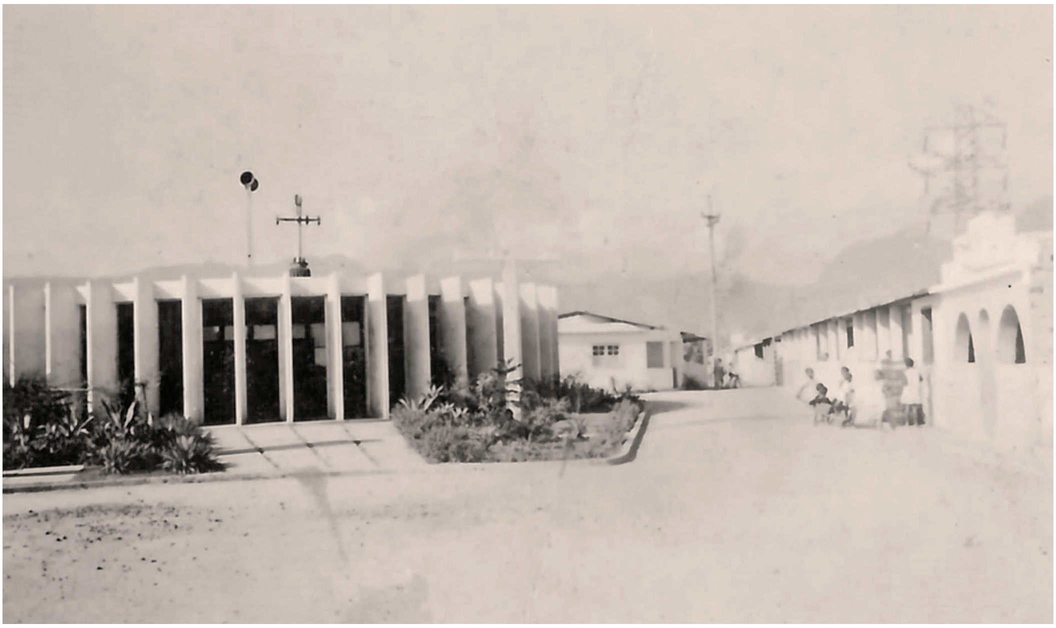

Figura 1: Igreja de São Daniel e seu entorno imediato, c.1960 (Acervo Luiz de Mello)

A arquitetura de Niemeyer uniria e abrigaria outros bens artístico-culturais, como o conjunto de quadros da via-sacra, pintado por Alberto da Veiga Guignard, especialmente para compor o templo; uma pia batismal (Figura 2), colonial autêntica, cedida pelo Serviço do Patrimônio Histórico e Artístico Nacional (Sphan); e uma réplica do profeta Daniel (Figura 3), reproduzida do modelo esculpido pelo artista Aleijadinho e que está em Congonhas do Campo (MG). O interior foi projetado pelo engenheiro decorador Heitor Coutinho – incluindo o desenho dos bancos, altar (Figura 3) e confessionário –, e o projeto paisagístico para o jardim do adro foi desenvolvido pelo engenheiro Paulo Athayde. O próprio Niemeyer, dias antes da inauguração, mostrou-se admirado com a obra, tendo feito os maiores elogios ao trabalho de Guignard e de Heitor Coutinho.

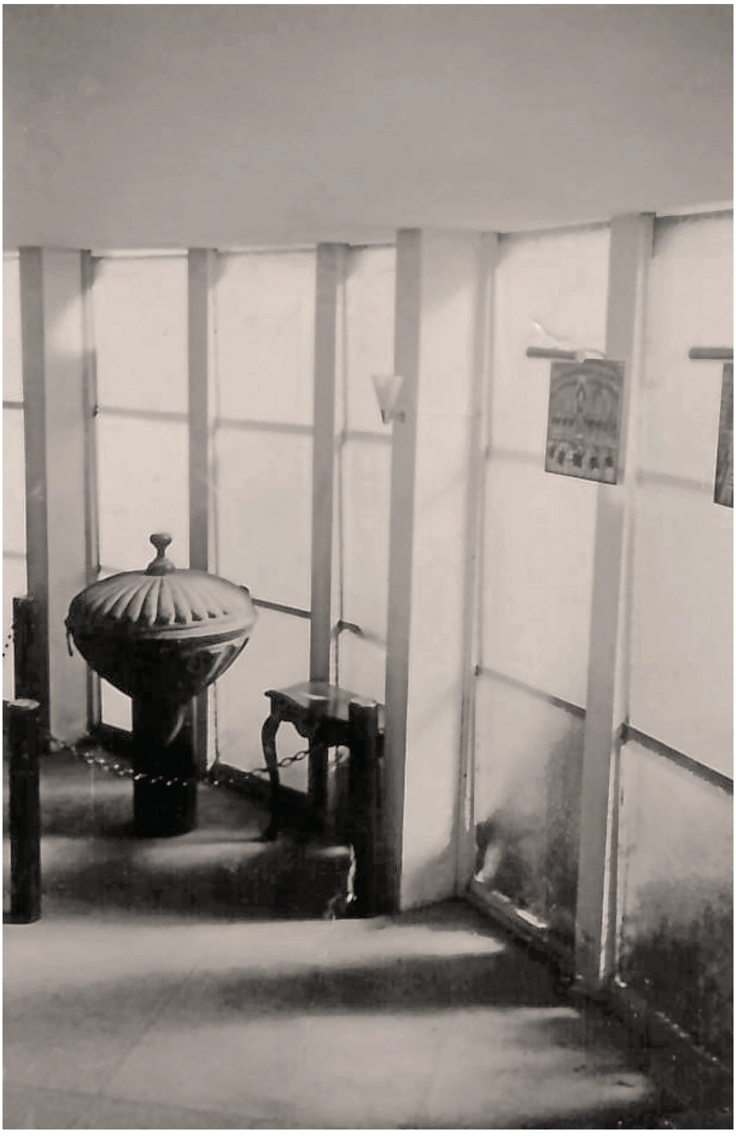

Figura 2: Interior do templo. Pia bastismal ao fundo e quadro da via-sacra, pintado por Guignard, fixado no pilar, c.1960 (Acervo Luiz de Mello)

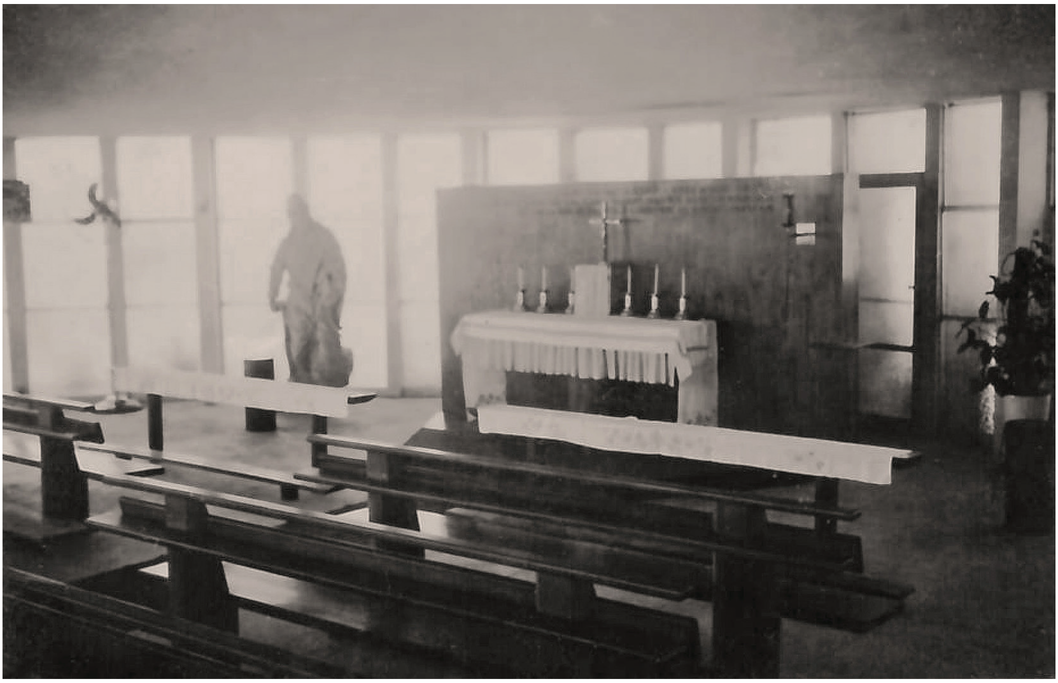

Figura 3: Interior do templo. Ao fundo, o altar e a réplica do profeta Daniel, moldado do original de Aleijadinho. Bancos em madeira, no primeiro plano, c.1960 (Acervo Luiz de Mello)

A igreja foi implantada no parque São José, um conjunto de habitações populares construído pela Fundação das Pioneiras Sociais (1956-1991),[2] destinado a funcionários da prefeitura (Fernandes, Costa, 2009). Teve grande protagonismo na proposta de construção da capela projetada pelo arquiteto Oscar Niemeyer a primeira-dama[3] do Estado, Elba Carvalho Sette Câmara, como parte do Programa de Recuperação do Parque São José.

Ela considerava ser providência de grande interesse, justificando ser uma demanda da própria comunidade, expressa no primeiro contato (Uma vida..., 13 set. 1960). Parte da sociedade ponderava que tal obra deveria ter sido realizada em outro local, sem a proximidade da favela (Igreja..., 19 out. 1960). Elba Sette Câmara, no entanto, argumentava não haver melhor local para a construção de uma capela tão maravilhosa. Sua localização seria intencional, uma vez que, "ficando em um parque proletário, os visitantes teriam a oportunidade de ver os verdadeiros problemas do povo carioca, sendo, portanto, mais generosos nas suas doações" (Mauro, 29 nov. 1960, p.3). Também considerava a possibilidade de atrair a atenção das autoridades e do povo para os problemas das favelas, pois, à medida que fossem visitar a obra, forçosamente poderiam sentir de perto os problemas intrínsecos ao entorno (Bela..., 3 dez. 1960).

O Parque São José seguia o modelo dos parques proletários que tinham por finalidade proporcionar as condições necessárias para uma "vida mais sadia" aos moradores das favelas, por meio de um controle social de caráter civilizatório que também envolve a questão sanitária, vinculando a saúde à habitação.

O Departamento de Assistência e Saúde da Prefeitura do Distrito Federal criou em 1943 o Serviço de Parques Proletários Provisórios (PPPs), sob a coordenação do médico sanitarista

Victor Tavares Moura (1882-1960), com o objetivo de substituir os espaços das favelas com a construção de habitações higiênicas. Na concepção de Moura (1940), era importante conter o "vício social" e a "promiscuidade" das favelas. Esse pensamento higienista, que une a desordem à transmissão de doenças, marcou o ambiente intelectual da virada do século XIX para o XX (Chalhoub, 2017).

É possível observar o caráter permanente desse conjunto, a partir das iniciativas conduzidas pelas primeiras-damas, senhora Alaísa Resende Sá Freire Alvim e, posteriormente, senhora Elba Carvalho Sette Câmara, com a implantação do Programa de Recuperação do Conjunto São José, que surge no contexto da oferta de "uma vida melhor para os habitantes dos núcleos residenciais do Estado" (Uma vida..., 13 set. 1960, p.2).

O Parque São José apresentava construções em alvenaria (Figura 1), que se aproximavam de um tipo de proposta de caráter mais permanente, distinto, portanto, de outros projetos de habitação popular da época. O conjunto era composto de 180 casas ocupadas por servidores da antiga prefeitura, cuja remuneração não ultrapassava um salário mínimo, situado em zona não urbanizada, onde residia numerosa população operária, incluindo favelados. Na época da inauguração, a paróquia contava com 1.300 habitantes (Mauro, 29 nov. 1960) e reunia, além dos moradores locais, fiéis de outros conjuntos populares oriundo de remoções de favelas e bairros próximos.

A iniciativa de construção do templo foi acordada com o monsenhor Bessa e tinha inteira aprovação do cardeal dom Jaime Câmara, que decidiu lhe dar o nome de são Daniel, o grande profeta, num ato de homenagem ao filho menor do casal Sette Câmara. Representou a primeira igreja dedicada ao santo profeta, em todo o mundo, "com intuito de começar mais uma devoção a um Santo de Deus, existente já há tantos séculos" (Programa-convite…, 25 jul. 1965, p.2). Dois dias após o início da construção já era noticiada a ideia de que a capela seria transformada em paróquia, segundo resolução do cardeal-arcebispo dom Jaime Câmara e comunicação feita pelo próprio a Elba Sette Câmara (Mauro, 16 set. 1960).

A construção do templo foi realizada em curto espaço de tempo, tendo sido iniciada em 14 de setembro de 1960 e concluída, em tempo recorde, em 28 de outubro de 1960. A imagem do profeta Daniel, única imagem dessa edificação, havia chegado, e, em poucos dias, a pia batismal também chegaria, ficando a inauguração do templo apenas na dependência da via-sacra que Guignard estava finalizando (Mauro, 29 out. 1960). Inaugurada em 2 de dezembro de 1960, só nas vésperas o governador Sette Câmara havia providenciado o asfaltamento do parque São José, tendo em vista a celebração.

Antes mesmo da inauguração, incluindo o fato de a capela já ter sido elevada à categoria de paróquia, já havia ocorrido a designação de um padre para estar à frente daquela comunidade, o vigário João Batista Petit (1960-1961). Na mesma ocasião foi iniciada uma ação de escotismo, formada por vinte meninos – os Escoteiros de São Daniel (Mauro, 29 nov. 1960) – e o Apostolado de Oração. A partir de 1961, foi designado o padre Julius Kremer para assumir a igreja.

A solenidade na inauguração da matriz de São Daniel da Praia Pequena contou com a presença do presidente da República, Juscelino Kubitschek (JK), sua esposa, Sara Kubitschek, e suas filhas, Márcia e Maria Estela, do governador provisório do estado da Guanabara, José Sette Câmara Filho, sua esposa, Elba Sette Câmara, do ex-prefeito Sá Freire Alvim, do ministro Paschoal Carlos Magno, do arcebispo dom Helder Câmara, do cardeal dom

Jaime de Barros Câmara e de outras altas autoridades civis, militares e eclesiásticas, além de centenas de fiéis (Bela..., 3 dez. 1960). Prevista inicialmente para as 11h, as solenidades de inauguração tiveram início precisamente às 10h, quando o presidente JK – cuja presença não estava prevista na programação oficial – desembarcou de um helicóptero da FAB (Figura 5) com sua esposa e foi aplaudido pela massa presente e saudado pelos alunos de escolas públicas formados em frente à igreja (Figura 6). Suas filhas chegaram no mesmo helicóptero alguns minutos depois (Bela..., 3 dez. 1960).

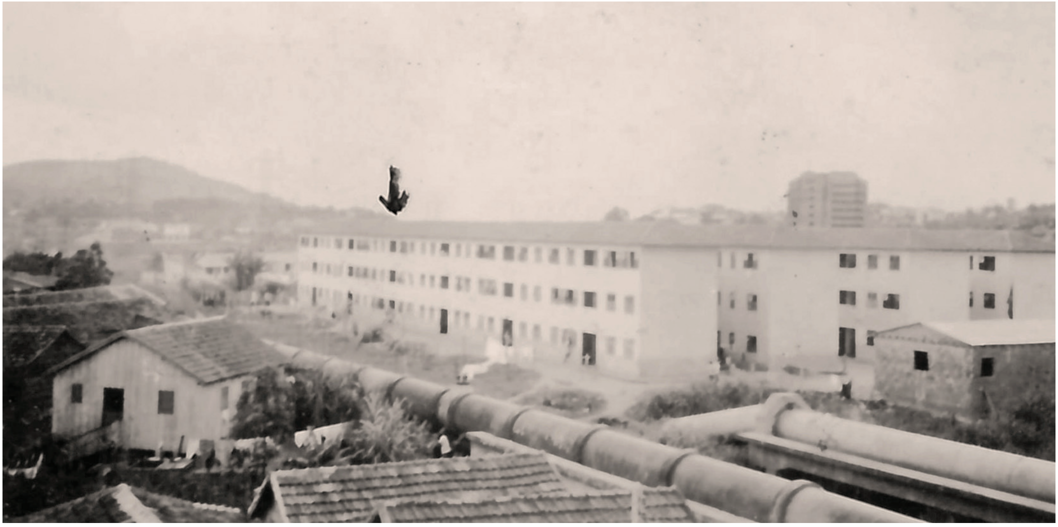

Figura 4: O vigário João Batista Petit passou a residir em um apartamento situado próximo à igreja, no terceiro pavimento. A seta, desenhada a caneta na fotografia original, aponta a localização, c.1960 (Acervo Luiz de Mello)

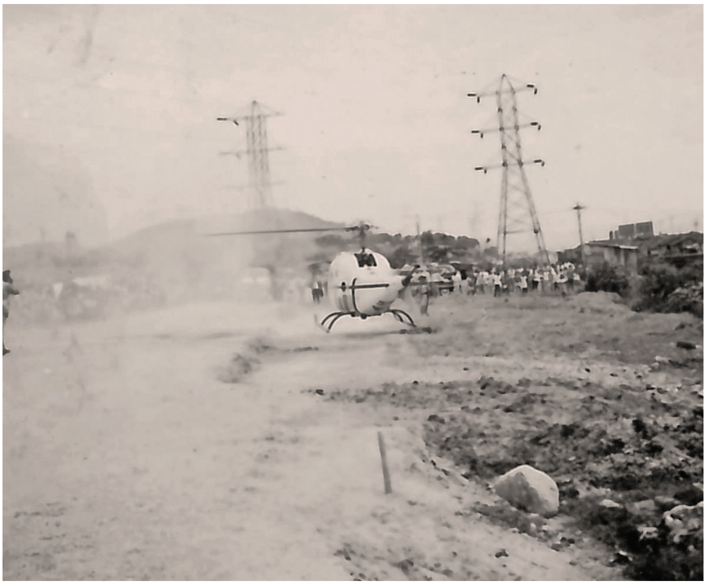

Figura 5: Pouso do helicóptero com comitiva presidencial, em área descampada nas proximidades da matriz de São Daniel, para a solenidade de inauguração do templo, 1960 (Acervo Luiz de Mello)

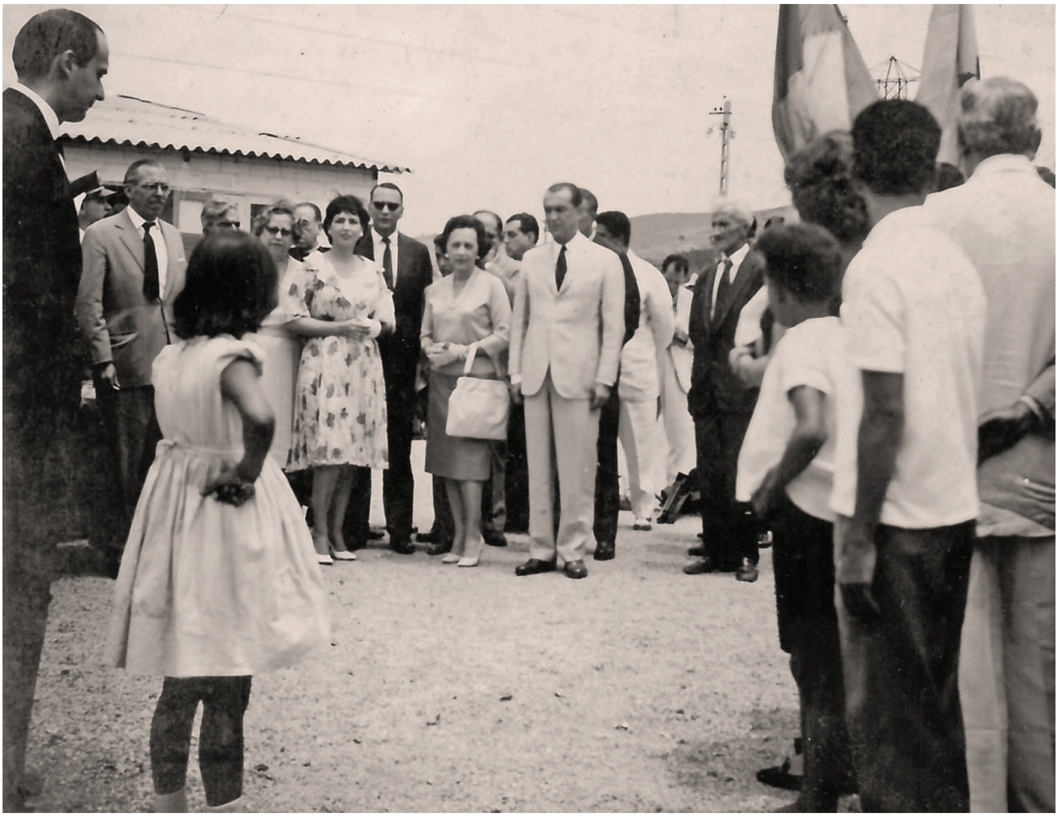

Figura 6: Inauguração da matriz de São Daniel, com destaque para a presença dos casais Kubitschek, Sette Camara e Sá Freire Alvim (do centro para à esquerda), 1960 (Acervo Luiz de Mello)

A construção da igreja teve grande envolvimento de pessoas próximas ao presidente JK (1956-1960), fato este que pode ser verificado nas imagens da coleção de Luiz de Mello. Pode-se destacar a atuação de José Sette Câmara Filho, que teve importante papel na diplomacia e na política brasileira, por anos acompanhando e colaborando em diversos momentos da trajetória política de JK. A construção da igreja esteve associada a esse contexto, quando foi nomeado pelo Senado Federal como governador-provisório do estado da Guanabara, no período de transição da capital federal para Brasília. Discretamente, a construção do templo foi movida por interesses políticos pessoais, considerando que não onerou o Estado, sendo totalmente custeada por particulares, reforçando o caráter filantrópico expresso no protagonismo das primeiras-damas.

A solenidade de inauguração contou com a bênção da edificação e da via-sacra pelo cardeal dom Jaime Câmara, auxiliado pelo arcebispo, e com o descerramento pelo presidente de uma placa de bronze alusiva ao ato. O cardeal (Figura 7), na ocasião, declarou que "estava grato ao governo por ter construído aquelas habitações e dado um pouco de conforto material àquela gente", mas enfatizava que "a obra estaria incompleta se a casa de Deus não se plantasse ali, pois a assistência espiritual e religiosa era mais importante que tudo o que se via em volta do santuário". Não faltou a algazarra das crianças, os acordes da banda de música (Figura 8) e o canto dos escoteiros, completando a festa, no lado de fora (Pinheiro, 3-4 dez. 1960, p.2).

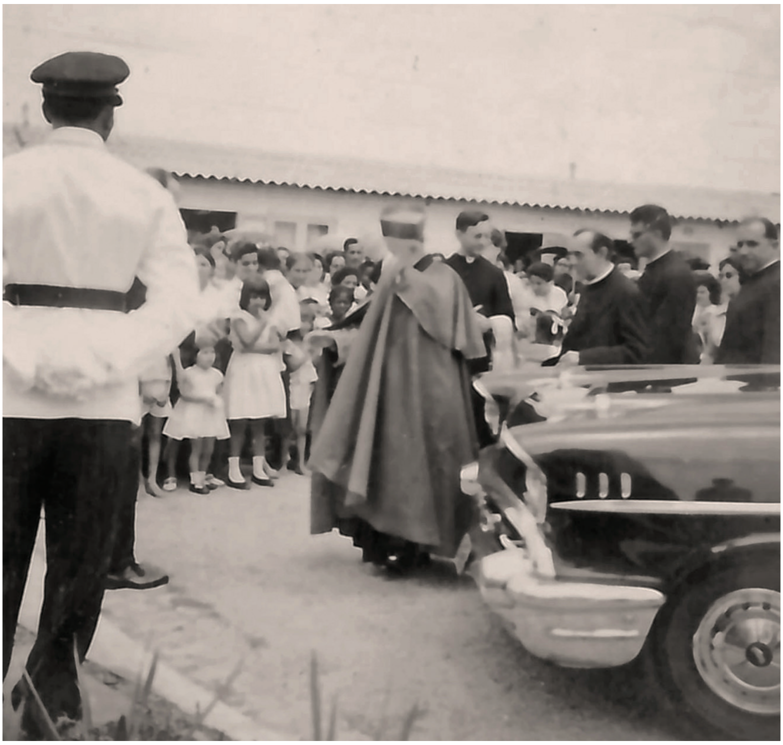

Figura 7: Chegada do cardeal dom Jaime Câmara para a solenidade de inauguração da matriz de São Daniel. Observa-se a multidão na recepção do sacerdote, 1960 (Acervo Luiz de Mello)

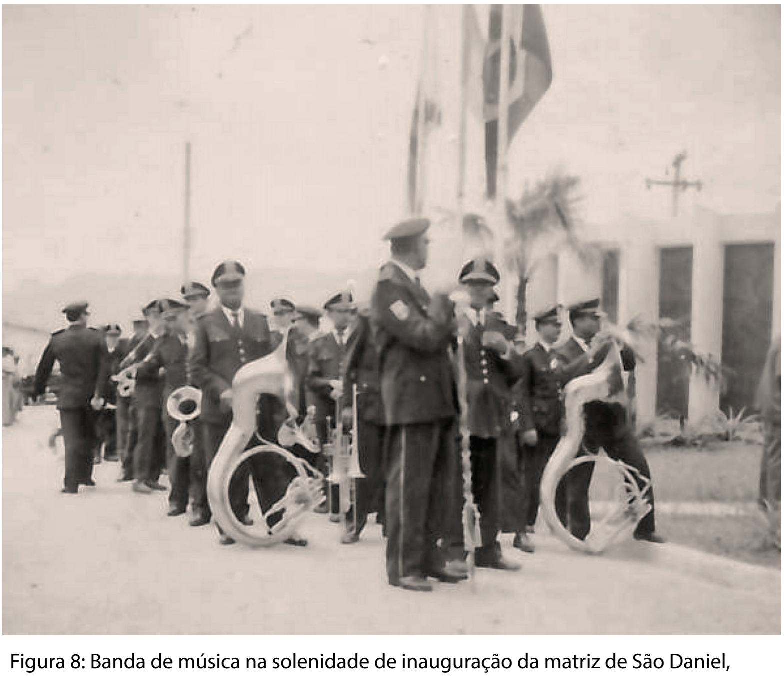

Figura 8: Banda de música na solenidade de inauguração da matriz de São Daniel, 1960 (Acervo Luiz de Mello)

Destaca-se o relato de antigas moradoras – Durvalina Augusta Costa e Luzia Camila Alves – registrado por Silva (2004), que associam a inauguração como um momento especial de sociabilidade e fortalecimento de laços comunitários.

> Um dia muito, muito bonito, com festas, bandas de música, fogos, onde todos participaram (Costa, 22 fev. 2004 citado em Silva, 2004, p.27).

> Foi muito bom, foi uma coisa grande, bonita, eu não sei explicar de tão bom que foi. A festa foi muito linda, fogos, música, tinha festa o mês inteiro. Aquilo alegrou os moradores, foi uma coisa muito linda. Tá vendo esse campo? Veio o Presidente de helicóptero (Alves, 15 fev. 2004 citado em Silva, 2004, p.28).

Nos anos que se seguiram à inauguração, a comunidade vivenciou uma intensa sociabilidade, por meio das atividades litúrgicas, sociais e culturais. Percebe-se nos registros fotográficos do período analisado a apropriação dos diferentes espaços da comunidade, para além do templo religioso, com atividades e festividades litúrgicas. Juntamente a outros conjuntos habitacionais, o parque São José passou para a administração da Fundação Leão XIII,[4] em 1962. O principal objetivo da entidade era "prestar ampla assistência social aos moradores dos morros, das favelas e de locais semelhantes na Cidade do Rio de Janeiro" (Brasil, 25 jan. 1947, p.1146), assumindo uma postura político-pedagógica voltada para fornecer assistência material e moral. Dentro do contexto político, a fundação tinha o objetivo de conter a mobilização operária de cunho esquerdista nas favelas.

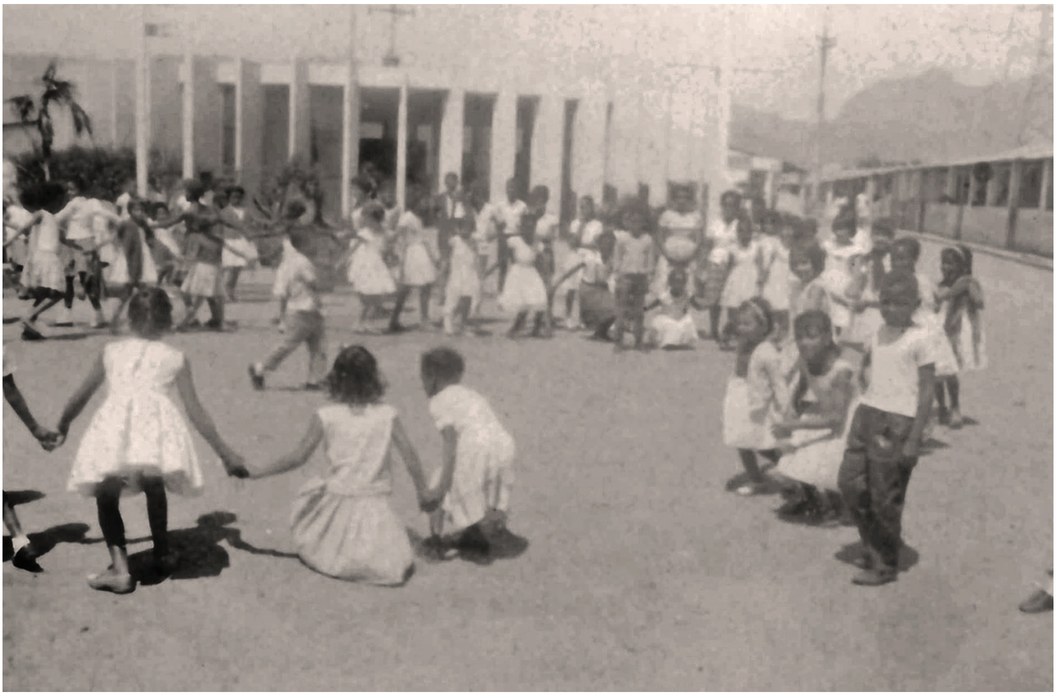

Figura 9: Alegres grupos de crianças em preparação para a primeira comunhão, no largo em frente ao templo, em 16 set. 1962 (Acervo Luiz de Mello)

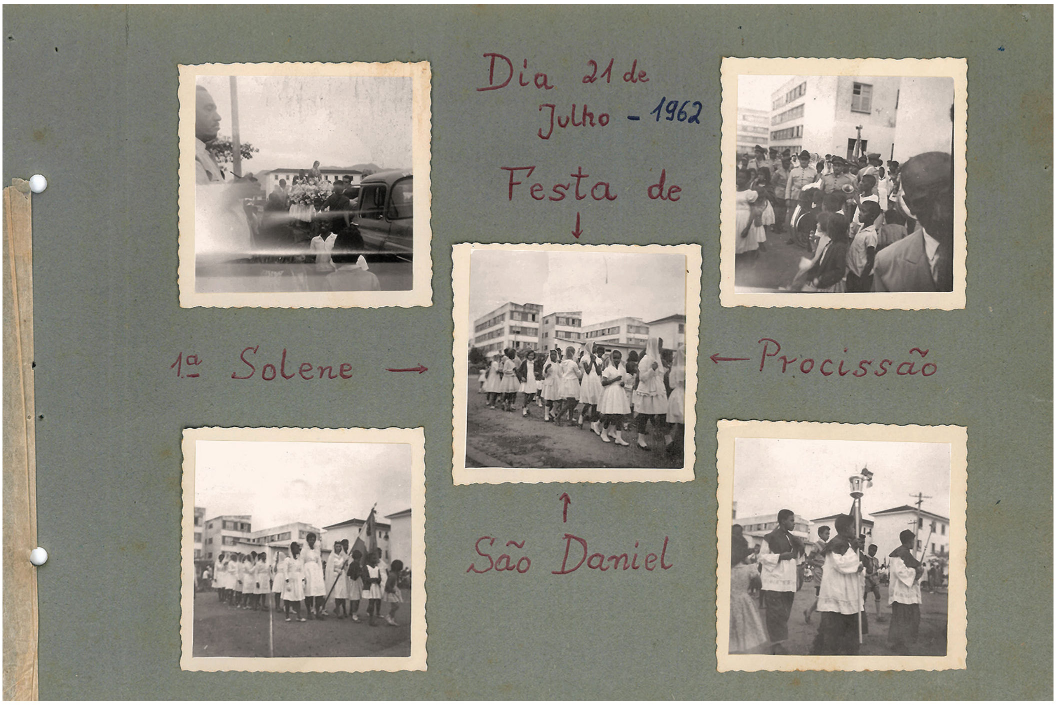

Figura 10: Página do álbum, contendo fotografias que representam a comunidade da matriz de São Daniel, em Marcha na Festividade do Padroeiro, em 21 de julho de 1963 (Acervo Luiz de Mello)

## Considerações finais

A edificação da igreja São Daniel Profeta ocorreu no período de fortes investimentos na habitação popular e no aumento das remoções conduzidas pelo poder público (1960-1970) na cidade do Rio de Janeiro. A Arquidiocese de São Sebastião do Rio de Janeiro teve papel importante na assistência social e formulação de propostas e políticas habitacionais, como setor conservador que se preocupava em impedir a ação do Partido Comunista nas favelas. A ação assistencialista da Igreja católica em favelas estava alinhada aos interesses dos poderes dominantes – o Estado, a Igreja católica e as elites econômicas do país em industrialização.

Para Oliveira (2014), no Rio de Janeiro o Serviço de Parques Proletários, juntamente com a Fundação Leão XIII e outras iniciativas, foi instituído dentro desse quadro ideológico. A autora ressalta que a política para as favelas não tinha verba definida e oscilava de acordo com os tempos e as iniciativas do governo federal. Nesse contexto, foi essencial a aliança entre Igreja e Estado que se estabeleceu entre 1945 e 1964. Assim, as ações em favelas eram vistas como favor, e não como um direito. Tais ações caridosas eram, geralmente, realizadas por mulheres da elite e vinculadas aos preceitos da Igreja católica de ajudar aos pobres.

Um pacto foi firmado pelas elites brasileiras em troca da promoção de alguns serviços para a sociedade (Burgos, 2006), como observamos no caráter filantrópico de construção da matriz de São Daniel, que buscavam amenizar o conflito social e afastar o perigo do comunismo, temor alimentado no contexto político instaurado a partir do Estado Novo.

Segundo Oliveira (2014), entre as décadas de 1940 e 1960, o principal eixo de ação do Estado no território de favelas ocorreu pela institucionalização de ações ligadas ao serviço social e à assistência social prestada indiretamente pelo poder público.

Não menos relevante, essa ação está associada a uma estratégia da Igreja católica em ocupar os mesmos territórios para apaziguar tensões sociais e promover a paz social. A participação da Igreja nas políticas habitacionais propiciou, segundo Burgos (2006), a cristianização das massas. Isto é, em vez do conflito político, prometia diálogo e compreensão; em vez da luta pelo acesso a bens públicos, o assistencialismo; no lugar da crítica, a resignação; em vez do intelectual orgânico, a formação de lideranças tradicionais.

Observa-se que os territórios exercem papel importante na construção de identidades e cidadania, e sob as análises sociológicas e geográficas e os aspectos políticos, econômicos, ambientais etc., a relação social nos territórios urbanos é marcada por disputas e conflitos, da mesma forma que o campo da memória social. O templo religioso e o conjunto de fotografias do álbum ancoram as memórias, fundamentam e reforçam os sentimentos de pertencimento do grupo.

## NOTAS

[1] O Grupo de Jovens, criado em 1974, teve atuação marcante na comunidade, com a mobilização política, social e recreativa de jovens dos bairros vizinhos, sendo sua maioria de Manguinhos. Após 1983, com a chegada do interventor, diversas atividades foram afetadas na igreja de São Daniel Profeta e paróquia de Santa Bernadete, assim como as atividades desse grupo, extinguidas em caráter de repressão política.

[2] A Fundação das Pioneiras Sociais foi uma instituição assistencial brasileira, fundada por iniciativa da ex-primeira-dama Sarah Kubitschek e reconhecida como instituição de utilidade pública à sociedade civil por meio do decreto n.39.865, de 29 de agosto de 1956, assinado pelo então presidente Juscelino Kubitschek. As ações voltavam-se principalmente para assistências médica e educacional da população pobre.

[3] A partir da década de 1940, as esposas dos chefes do Executivo passam a ser designadas primeiras-damas e a exercer atividades de assistência social, voltadas para o plano da atenção à pobreza, em ordem filantrópica. O marco dessa prática teve protagonismo de Darcy Vargas, em 1942, com a constituição da Legião Brasileira de Assistência (LBA).

[4] Em 1946, a prefeitura do Distrito Federal entra em entendimento com o cardeal dom Jaime de Barros Câmara, da Arquidiocese do Rio de Janeiro, para criação da Fundação Leão XIII, por meio do decreto presidencial n.22.498 de 22 de janeiro de 1947.
